# Successful Treatment of Refractory Massive Pulmonary Embolism with Repeated Administration of Systemic Thrombolysis

**Published:** 2018-02

**Authors:** Armeen D. Poor, Hooman D. Poor

**Affiliations:** Division of Pulmonary, Critical Care, and Sleep Medicine, Icahn School of Medicine at Mount Sinai, New York, USA

**Keywords:** Acute pulmonary hypertension, Alteplase, Massive pulmonary embolism, Obstructive shock, Right ventricular failure, Systemic thrombolysis

## Abstract

**Background:**

We report a case series of two patients in the intensive care unit with massive pulmonary embolism and obstructive shock who had resolution of shock after repeated administration of alteplase.

**Case Summaries:**

Both patients were initially dosed 10 mg of alteplase followed by infusion of 90 mg over 2 hours, but remained in obstructive shock requiring significant inotropic and vasopressor support. Both patients were deemed poor candidates for embolectomy. The first patient received repeated doses reaching an accumulative dose of 200 mg alteplase over 15 hours. The second patient received an accumulative dose of 250 mg alteplase over 36 hours. Both patients had resolution of shock within 24 hours of repeated administration of alteplase, but also experienced significant drops in hemoglobin, which were supported with transfusions. They were transferred out of the intensive care unit after resolution of obstructive shock and hemorrhage. The first patient died one week after transfer from the intensive care unit due to invasive candidiasis and septic shock. The second patient was weaned from the ventilator and discharged home.

**Conclusion:**

Patients with obstructive shock secondary to massive pulmonary embolism despite a one-time dose of alteplase and poor candidacy for embolectomy may benefit from repeated doses of alteplase. Due to the short half-life, repeated administration of thrombolytic may be appropriate for younger patients without absolute contraindications to thrombolysis, but future studies are needed to identify the optimal patient population.

## INTRODUCTION

Pulmonary embolism (PE) is associated with significant in-hospital morbidity and mortality. Massive PE, defined as sustained hypotension with systolic blood pressures less than 90 mmHg for at least 15 minutes or requiring inotropic support, without evidence of other etiologies for shock, has a poor prognosis with in-hospital mortality reported as high as 52% (1). Guidelines from medical societies recommend thrombolysis for massive PE, but there is limited evidence to support the superiority of any particular dosing regimen (2–4). The recommended dosing of thrombolytic agents, most commonly tissue plasminogen activator (tPA), is derived from dated clinical trials evaluating the use of tPA for patients with acute myocardial infarction (AMI) (5–7). To our knowledge, repeated administration of thrombolytics in the setting of massive PE, too unstable for surgical embolectomy, has only been reported in patients with cardiac arrest (8–10), but may be beneficial for patients with persistent obstructive shock. We report two patients with massive PE with complete resolution of shock after multiple repeated administration of tPA with high 24-hour cumulative doses of tPA in the absence of cardiac arrest..

## CASE SUMMARIES

### Case One

A 26-year-old woman with a history of metastatic mucinous adenocarcinoma of the appendix presented to the hospital with abdominal pain. A large volume paracentesis removed 5 liters of fluid. Shortly after the procedure the patient experienced respiratory distress, followed by a cardiac arrest. Return of spontaneous circulation was achieved after 7 minutes of CPR. Upon return of spontaneous circulation, the patient’s blood pressure was 89/66 mmHg with a heart rate of 145 beats per minute. Bedside transthoracic echocardiogram (TTE) revealed a severely dilated right ventricle and a large right atrial thrombus. Labs were notable for a venous blood gas with pH 7.08, pCO2 56 mmHg, in addition to troponin 1.61 ng/mL. She received 10 mg of alteplase followed by a 90 mg infusion over 2 hours. She was transferred to the intensive care unit (ICU) receiving 30 mcg/min of norepinephrine and 7.5 of mcg/kg/min dobutamine, but remained persistently tachycardic and hypotensive upon arrival. She had continued evidence of severe right ventricular (RV) dilatation on TTE, cold extremities on physical exam, and a central venous oxygen saturation of 22%. She was deemed a poor surgical candidate by a multidisciplinary PE team. Given her persistent severe obstructive shock 6 hours later, requiring 20 mcg/min of norepinephrine and 7.5 mcg/kg/min of dobutamine, another 50 mg of alteplase was administered over 4 hours. Four hours after completion of the second alteplase infusion, the patient remained in severe obstructive shock requiring high doses of vasopressors and inotropes and an additional 50 mg of alteplase was administered over 2 hours. Overall, the patient received a total of 200 mg of tPA within a 15-hour period. Her course became further complicated by an acute drop in hemoglobin due to hemoperitoneum, with an initial 2.0 g/dL drop in Hb from 8.0 to 6.0 g/dL, occurring 16 hours after the initial tPA infusion. She was supported with a total of 10 units packed red blood cells over the next 48 hours. She was then weaned off all vasopressors and inotropes, and underwent IR-guided embolization of the right hepatic artery, after which her hemoglobin remained stable. A subsequent TTE demonstrated significant improvement in RV function. Four days later she was successfully extubated and transferred out of the ICU. Her course was ultimately complicated by aspiration pneumonia and septic shock secondary to invasive candidiasis; she expired one week later.

### Case Two

A 46-year-old man with history of asthma was brought into the hospital by ambulance for respiratory distress. He was initially managed in the ICU for status asthmaticus requiring heavy sedation and paralysis. His asthma improved, but his course was complicated by bilateral segmental PE detected on computed tomography angiography (CTA). TTE revealed mild RV dilatation and normal RV function. He was treated with low molecular weight heparin and eventually received a tracheostomy because of respiratory weakness, likely from critical illness polymyoneuropathy. While out of the ICU, he underwent placement of a percutaneous endoscopic gastrostomy (PEG) tube, for which anticoagulation was held for one day. The day after PEG placement the patient became acutely hypotensive, with systolic blood pressures between 70 and 80 mmHg. He received a 2 liter normal saline bolus, after which he suffered an asystolic cardiac arrest. ROSC was achieved after 6 minutes of CPR and he was transferred back to the ICU. His arterial blood gas was notable for a pH of 7.05 and pCO2 77 mmHg. He required 20 mcg/min of norepinephrine, 2.4 mL/hr of vasopressin, 300 mcg/min of phenylephrine, 5 mcg/kg/min of dobutamine, and 40 ppm inhaled nitric oxide. TTE demonstrated severe decreased RV function with bulging of the interventricular septum into left ventricle. Due to high suspicion for massive PE, 10 mg of alteplase was administered, followed by a 90 mg infusion over 2 hours. 18 hours later he had continued evidence of severe RV strain on bedside TTE, including unchanged vasopressor and inotropic support. He received 3 more doses of 50 mg of alteplase, administered over 2 hours each and 3 hours apart. In total the patient received 250 mg of alteplase in a 36-hour period. The next day inotropes and inhaled nitric oxide were weaned off. Pulmonary angiography revealed normal pulmonary artery pressures and no clot. TTE demonstrated mild decreased RV function. His course was further complicated by melena and anemia, which stabilized after transfusion of 2 units packed red blood cell, 1 unit platelets, and 2 units fresh frozen plasma. In the setting of persistent altered mental status, the patient underwent an MRI which showed evidence of embolic infarcts; a transesophageal echocardiogram (TEE) revealed a patent foramen ovale, which was later closed percutaneously. His mental status returned to baseline and he was weaned off the ventilator. He ultimately underwent decannulation of his tracheostomy, removal of his PEG tube, and was discharged home.

## DISCUSSION

The management of massive PE poses many challenges to providers. Guidelines recommend the administration of thrombolytic therapy when massive PE is suspected, but few offer substantial evidence for dosing (2–4). Patients with massive PE present with obstructive shock as abrupt elevations in afterload impair RV contractility and cause RV dilatation with bowing of the interventricular septum, impeding LV diastolic filling and ultimately reducing cardiac output. Worsening tricuspid regurgitation and RV ischemia secondary to RV wall stress and reduced right coronary artery perfusion also exacerbate this process (4). The mortality of patients with obstructive shock secondary to PE is high, and treatment with thrombolytic agents to relieve the obstructive etiology is recommended (6, 11, 12). The patient in Case 1 had thrombus visualized in the right atrium in the setting of acute RV dilatation and shock, while the patient in Case 2 had a history of known PE with high suspicion of obstructive physiology due to evidence of acute right heart failure and shock after brief cessation of anticoagulation. Both benefited from the repeated administration of thrombolytic therapy.

Tissue plasminogen activator (tPA) is a protease enzyme that binds to fibrin and activates the conversion of plasminogen to plasmin, a potent fibrinolytic that facilitates rapid clot dissolution (13). Randomized controlled trials comparing thrombolytic therapy in massive PE to other forms of anticoagulation PE are lacking and likely not feasible. A meta-analysis by Wan et al reported a significant risk reduction of death or recurrent PE from 19.0% with heparin to 9.4% with fibrinolysis for massive PE (1). However, the recommended dosing of tPA in massive PE is not consistent across consensus guidelines. The American Heart Association (AHA) recommends a 2-hour infusion of 100 mg alteplase (Class IIa, level of evidence B) (2), the British Thoracic Society (BTS) recommends a 50 mg bolus (Grade C) (3), and the European Society of Cardiology recommends either 100 mg tPA over 2 hours or 0.6 mg/kg over 15 minutes (4). The current dosing for tPA has been predominantly derived from earlier studies evaluating the role of fibrinolysis for acute MI. A 1988 randomized controlled trial compared tPA to urokinase in the treatment of acute PE, using a 2-hour dose of tPA (5). This dosing was carried over from an earlier trial regarding coronary thrombolysis in acute MI (7). The FDA-recommended dose reflects these trials, limiting the dose of tPA to 100 mg over 2 hours. While lower doses of tPA as compared to standard doses have been studied, there are only case reports of higher doses of tPA for massive PE, predominantly in the context of cardiac arrest (8–10). A 2006 single-center prospective registry of patients with acute PE noted a trend towards improved mortality and reduced bleeding when comparing rescue embolectomy to repeat thrombolysis, but a large majority of these patients were not in shock (14). The functional half-life of tPA is approximately 3 minutes, and thus the essential fibrinolytic activity of the medication is gone soon after the infusion is complete (15). One pharmacokinetic study evaluating higher dose of tPA in acute MI found that double-bolus doses of 50 mg, administered 30 minutes apart, resulted in therapeutic ranges of tPA for up to 90 minutes (16). Considering the short half-life of tPA, patients who continue to have severe obstructive shock secondary to clot may benefit from re-administration of fibrinolytic if initial dosing does not sufficiently lyse the clot. In our experience, many patients with massive PE continue to have significant residual clot after receiving tPA as can be demonstrated when undergoing surgical thrombectomy or repeat CT angiography.

The risk of bleeding with tPA is not insignificant, with reports of major bleeding ranging from 9.1 to 11.5% (1, 6, 17, 18). Studies have shown that older patients may be further predisposed to bleeding complications (16, 17), and perhaps as in our cases, younger patients without absolute contraindications to thrombolysis may have a more favorable risk-benefit ratio. In the PEITHO trial, in which patients with submassive PE were given tenecteplase, only 4.1% of patients under 75 years old experienced major bleeding, versus 11.1% of those older than 75 (17). Older patients may also be predisposed to higher risk with extended thrombolysis, as demonstrated by Stangl et al, who found stronger fibrinolytic properties among patients older than 63-years-old who received double-bolus doses (16). As a result, in younger patients who are in shock requiring high doses of inotropic and vasopressor medications, too unstable for surgical intervention, repeated doses of tPA may be considered. Both of our patients experienced significant bleeding that was quickly stabilized, but overall they benefited from the hemodynamic improvement as a result of repeated thrombolysis. The BTS recommends fibrinolytic therapy ‘on clinical grounds alone if cardiac arrest is imminent’ (3) – we suggest that perhaps in younger patients without absolute contraindication to thrombolysis, with severe, persistent obstructive shock despite thrombolysis with 100 mg of tPA, there may be a role for repeated lysis beyond standard dosing. Further research is needed to identify an appropriate subset of patients with massive PE in whom repeated dosing of fibrinolytic may pose greater benefit than risk and optimal dosing regimens.
